# SGLT2 Inhibitors in MASLD (Metabolic Dysfunction-Associated Steatotic Liver Disease) Associated with Sustained Hepatic Benefits, Besides the Cardiometabolic

**DOI:** 10.3390/ph18081118

**Published:** 2025-07-26

**Authors:** Mohamad Suki, Ashraf Imam, Johnny Amer, Yael Milgrom, Muhammad Massarwa, Wadi Hazou, Yariv Tiram, Ofer Perzon, Yousra Sharif, Joseph Sackran, Revital Alon, Nachum Lourie, Anat Hershko Klement, Safa Shibli, Tamer Safadi, Itamar Raz, Abed Khalaileh, Rifaat Safadi

**Affiliations:** 1 Institution of Liver Diseases, Hadassah Medical Organization, Hadassah-Hebrew University, Jerusalem 91120, Israel; joni@hadassah.org.il (J.A.); yaelmil@hadassah.org.il (Y.M.); mohamadm@hadassah.org.il (M.M.); wadiha@hadassah.org.il (W.H.); yarivti@hadassah.org.il (Y.T.); oferper@hadassah.org.il (O.P.); yousras@hadassah.org.il (Y.S.); joseph@hadassah.org.il (J.S.); arevital@hadassah.org.il (R.A.); nachum@hadassah.org.il (N.L.); anat.klement@gmail.com (A.H.K.); safashibli8@gmail.com (S.S.); tamersafadiw@gmail.com (T.S.); razitamar502@gmail.com (I.R.); safadi@hadassah.org.il (R.S.); 2 Transplantation Unit, Department of Surgery, Hadassah Medical Organization, Hadassah-Hebrew University, Jerusalem 91120, Israel; achrafim@hadassah.org.il (A.I.); hbedk@hadassah.org.il (A.K.)

**Keywords:** SGLT2 inhibitors, NAFLD, MASLD, liver outcomes, cirrhosis, mortality

## Abstract

**Background and Aims**: Sodium-glucose cotransporter-2 (SGLT2) inhibitors have shown promise in metabolic dysfunction-associated steatotic liver disease (MASLD). This large real-world study aimed to evaluate the effects of SGLT2 inhibitors on MASLD patients’ clinical outcomes and liver-related complications over extended follow-up. **Patients and Method**: Data were sourced from TriNetX, a global health research platform with de-identified electronic medical records spanning 135 million patients across 112 healthcare organizations worldwide. We included MASLD adults diagnosed according to ICD9/10 criteria. Following propensity score matching based on 34 variables (demographics, comorbidities, laboratory tests and medication history), SGLT2 inhibitor-treated (n = 19,922) patients were compared with non-SGLT2 inhibitor (n = 19,922) cases. Exclusion criteria included baseline improved alanine aminotransferase (ALT) and alkaline phosphatase (ALP) levels > 4 upper normal limit (UNL), baseline advanced liver disease, liver transplant and cancer, past anticoagulation and non-MASLD etiologies. Assessed outcomes included survival, biochemical, hematologic, AFP, metabolic and cardiovascular parameters, progression to advanced liver disease (ALD), synthetic function, and metabolic markers over 1, 5, and 10 years. **Results**: Following matching, both cohorts were well-balanced across baseline characteristics. After one year, the SGLT2 inhibitor group demonstrated significantly reduced BMI (33.2 ± 6.2 vs. 34.1 ± 6.5 kg/m^2^, *p* < 0.001), improved ALT (40.3 ± 31.5 vs. 48.3 ± 41.2 U/L, *p* < 0.001), and better glycemic control (HbA1c 7.35 ± 1.51% vs. 7.93 ± 1.72%, *p* < 0.001). The SGLT2 inhibitor group showed higher 10-year survival rates (95.00% vs. 88.69%, *p* < 0.001), fewer cardiovascular events (10.19% vs. 11.80%, *p* < 0.001), and markedly reduced progression to advanced liver disease (6.90% vs. 14.15%, *p* < 0.001). These benefits were consistent across clinical, laboratory, and medication-defined ALD categories. Notably, rates of hepatic decompensation events were significantly lower with SGLT2 inhibitor therapy. **Conclusions**: In this large real-world cohort, SGLT2 inhibitor use in MASLD patients was associated with significantly improved long-term survival, cardiovascular, and liver-related outcomes over 10 years of follow-up. These benefits likely result from combined metabolic improvements, anti-inflammatory effects, and direct hepatoprotective mechanisms. SGLT2 inhibitors represent a promising therapeutic strategy for improving outcomes in MASLD.

## 1. Introduction

Metabolic dysfunction-associated steatotic liver disease (MASLD), previously known as non-alcoholic fatty liver disease, represents a significant global health burden, affecting approximately 25–30% of the world’s population and increasingly becoming the leading cause of chronic liver disease worldwide [1,2]. MASLD encompasses a spectrum of conditions ranging from simple steatosis to metabolic dysfunction-associated steatohepatitis (MASH), which can progress to advanced fibrosis, cirrhosis, and hepatocellular carcinoma (HCC) [3]. The disease is closely linked to metabolic syndrome components, including obesity, type 2 diabetes mellitus (T2DM), dyslipidemia, and hypertension, which collectively contribute to its pathogenesis and progression [4,5].

The global prevalence of MASLD continues to rise in parallel with the obesity epidemic, with projections indicating that the number of individuals with advanced-stage disease will double by 2030 [6,7]. Among patients with T2DM, the prevalence of MASLD reaches 50–75%, with approximately 17% developing advanced liver fibrosis [8]. The risk of disease progression to advanced liver disease, including liver cancer, is significantly higher in MASLD patients with diabetes compared to those without, highlighting the importance of early intervention in this high-risk population [9].

Despite its high prevalence and potential for serious complications, therapeutic options for MASLD remain limited. Current management strategies primarily focus on lifestyle modifications, including weight loss through diet and exercise, which have shown efficacy but are often difficult to maintain long-term [10]. While several pharmacotherapies have been investigated, including vitamin E, pioglitazone, and obeticholic acid, until recently, only resmetirom had received regulatory approval specifically for MASLD treatment, being granted accelerated FDA approval in March 2024 for noncirrhotic MASH with moderate to advanced liver fibrosis [11,12].

Sodium-glucose cotransporter-2 (SGLT2) inhibitors, initially developed for T2DM management, have emerged as a promising therapeutic class for MASLD due to their pleiotropic metabolic and cardiovascular benefits [13]. These agents, including empagliflozin, dapagliflozin, canagliflozin, and ertugliflozin, work by inhibiting glucose reabsorption in the proximal renal tubules, leading to glucosuria and subsequent improvements in glycemic control, weight reduction, and blood pressure [14]. Beyond their glucose-lowering effects, SGLT2 inhibitors have demonstrated remarkable cardiovascular and renal benefits in landmark trials, leading to expanded indications beyond diabetes management [15,16].

The potential hepatoprotective effects of SGLT2 inhibitors have garnered increasing interest. Preclinical studies have shown that these agents reduce hepatic steatosis, inflammation, and fibrosis through multiple mechanisms, including enhanced fatty acid oxidation, reduced de novo lipogenesis, decreased oxidative stress, and modulation of inflammatory pathways [17,18]. Clinical studies have reported improvements in liver enzymes, reduction in liver fat content measured by imaging, and favorable changes in non-invasive fibrosis markers in MASLD patients treated with SGLT2 inhibitors [19,20].

Recent meta-analyses of randomized controlled trials have provided evidence supporting the efficacy of SGLT2 inhibitors in improving hepatic steatosis and fibrosis in MASLD patients, with consistent benefits observed across different agents within the class [21,22]. The EMPA-REG OUTCOME, CANVAS, and DECLARE-TIMI 58 trials demonstrated cardiovascular benefits that extended to MASLD subpopulations, suggesting potential for reducing both hepatic and cardiovascular complications in these high-risk patients [23,24,25].

However, most clinical trials have been limited by relatively short follow-up periods, small sample sizes, and carefully selected patient populations that may not reflect real-world clinical practice. Real-world evidence is crucial to supplement clinical trial data, particularly for complex conditions like MASLD, where patient heterogeneity and comorbidities can significantly impact treatment outcomes [26]. Real-world studies offer insights into medication effectiveness and safety in diverse patient populations, including those typically excluded from clinical trials.

The present study aimed to evaluate the impact of SGLT2 inhibitor therapy on liver-related outcomes and overall survival in a large, real-world cohort of MASLD patients followed for up to 10 years. Utilizing the TriNetX global database and extensive propensity score matching, we sought to provide robust evidence on SGLT2 inhibitors’ effectiveness in modifying MASLD progression and improving patient outcomes in routine clinical practice. To our knowledge, this represents the largest and most comprehensive real- world evaluation of SGLT2 inhibitors in MASLD to date, with extended follow-up to assess long-term outcomes across multiple clinical domains.

## 2. Results

### 2.1. Baseline Characteristics

The matched cohort included 19,922 SGLT2 inhibitor-exposed and 19,922 SGLT2 inhibitor-unexposed MASLD patients. Baseline characteristics are presented in Table 1a,b, covering demographics, comorbidities, and laboratory parameters. Rigorous propensity score matching across 34 variables minimized confounding, ensuring comparability between groups.

### 2.2. Demographics and Comorbidities (Table 1)

Both cohorts were well-matched for demographics and comorbidities, as the data were available for all included participants. Mean ages, gender rates, race distribution, mean BMI, and prevalence of metabolic comorbidities (diabetes mellitus, hypertension, and ischemic heart disease) are completely matched between the study groups. Both groups are showing predominance of males and a White racial background. The baseline BMI of 34.0 ± 6.4 kg/m^2^ in the SGLT2 inhibitor group and 34.1 ± 6.5 kg/m^2^ in the non-SGLT2 inhibitor group confirms the predominantly obese population. The cohort had high rates of metabolic comorbidities, including diabetes mellitus (90.4%) and hypertension (66.7%). Both MASLD groups had 9.6% non-diabetic patients, suggesting a non-diabetic indication for SGLT2 inhibitor admonition. The SGLT2 inhibitor indication could be either cardiovascular or renal disease. The presence of established cardiovascular disease was evident with 11.1% in the SGLT2 inhibitor group versus 11% in the non-SGLT2 inhibitor group having ischemic heart disease and 2.6% with cerebrovascular disease.

### 2.3. Laboratory Parameters (Table 1)

Baseline laboratory values reflected the metabolic nature of the cohort. Mean liver enzymes (ALT, AST, ALP, GGT, and total bilirubin), creatinine, platelet count, INR, and AFP were similar in both groups, with >70% data availability for ALT and AST and 69% for total bilirubin and Platelet count, suggesting a strong database for comparison. The creatinine similarity is also standardizing the renal non-diabetic indication for the SGLT2 inhibitor. Metabolic parameters showed a similar poor glycemic control (increased HbA1c) and dyslipidemia (LDL, HDL, and TG showing hypertriglyceridemia) in both groups, with 62–70% data availability.

### 2.4. Clinical Outcomes

Table 2 summarize the central results regarding the impact of SGLT2 inhibitor exposure on major clinical outcomes, laboratory data, and categorical laboratory outcomes.

### 2.5. Mortality and Survival (Table 2, Figure 1)

**Figure 1:**
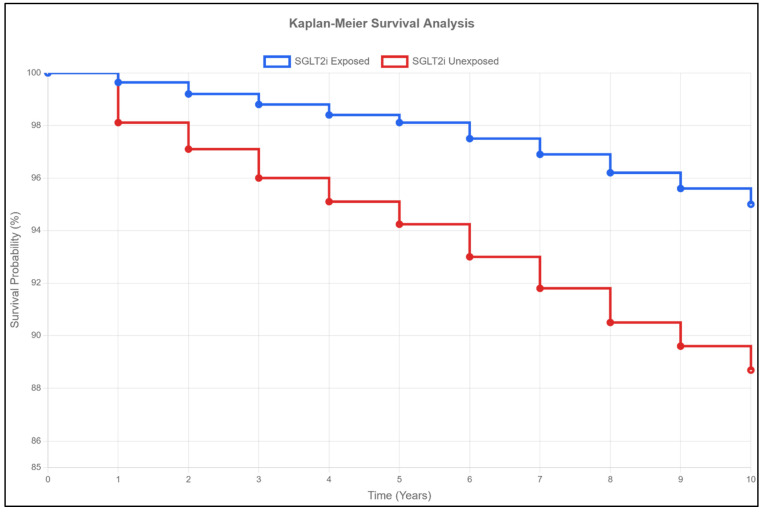
**Kaplan–Meier Survival Curves.** This figure presents the Kaplan–Meier survival analysis comparing the long-term survival of propensity score-matched MASLD patients with and without SGLT2 inhibitor exposure over a 10-year follow-up period. The survival rates at 10 years were 95.0% for SGLT2-exposed patients versus 88.7% for unexposed patients (*p* < 0.001). SGLT2i = SGLT2 inhibitor.

SGLT2 inhibitor therapy was associated with substantial mortality and survival benefits. Mortality rates were consistently lower in the SGLT2 inhibitor group across all time points: 0.30% vs. 1.63% at 1 year, 0.87% vs. 3.21% at 5 years, and 1.06% vs. 3.75% at 10 years (all *p* < 0.001). Survival rates were also favorable in the SGLT2 inhibitor group, being 99.6% vs. 98.1% at 1 year, 98.1% vs. 94.2% at 5 years, and 95.00% versus 88.69% at 10 years (all *p* < 0.001). The Kaplan–Meier survival curve (Figure 1) demonstrates this persistent survival advantage throughout the extended follow-up period.

**Figure 2:**
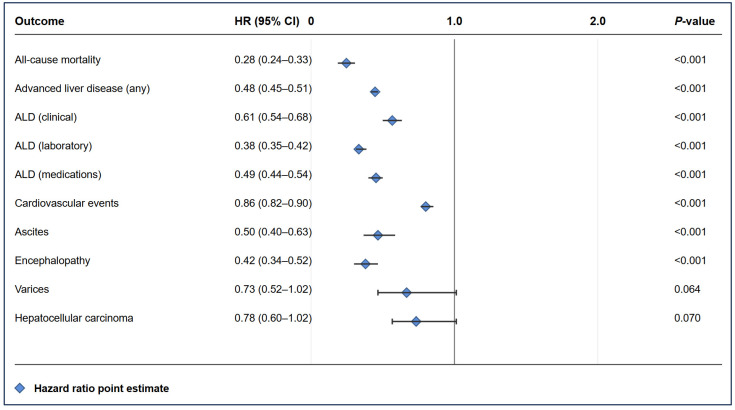
**Forest Plot of Hazard Ratios (SGLT2 inhibitors vs. Non-SGLT2 inhibitors)** This forest plot displays the hazard ratios and 95% confidence intervals for all major clinical outcomes, comparing SGLT2 inhibitor-exposed patients to unexposed patients. Key findings include significant risk reductions for mortality (HR 0.28), advanced liver disease (HR 0.48), and liver-related complications.

The calculated hazard ratio for all-cause mortality was 0.28 (95% CI 0.24–0.33), indicating a 72% reduced risk of death with SGLT2 inhibitor therapy (Figure 2). The number needed to treat to prevent one death was 74 at 1 year, decreasing to 36 at 10 years, reflecting cumulative benefits over time (Table 3).

### 2.6. Cardiovascular Events (Table 2)

Cardiovascular event rates were significantly lower with SGLT2 inhibitor therapy. At 10 years, cardiovascular events occurred in 10.19% of SGLT2 inhibitor patients versus 11.80% of controls (*p* < 0.001).

The hazard ratio was 0.86 (95% CI 0.82–0.90), representing a 14% risk reduction. While this benefit was not significant at 1 year (5.99% vs. 6.19%, *p* = 0.390), it became apparent by 5 years (9.50% vs. 10.28%, *p* = 0.009).

### 2.7. Advanced Liver Disease (Table 2, Figure 3)

SGLT2 inhibitor therapy markedly reduced progression to advanced liver disease. At 10 years, any ALD was present in 6.90% of SGLT2 inhibitor patients versus 14.15% of controls (*p* < 0.001), representing a 52% relative risk reduction (HR 0.48, 95% CI 0.45–0.51). This protection was consistent across all ALD categories:

- Clinical ALD: 2.20% vs. 3.60% at 10 years (HR 0.61, 95% CI 0.54–0.68).
- Laboratory-defined ALD: 3.10% vs. 8.10% at 10 years (HR 0.38, 95% CI 0.35–0.42).
- ALD requiring medications: 3.10% vs. 6.30% at 10 years (HR 0.49, 95% CI 0.44–0.54).

The NNT to prevent one case of advanced liver disease was 17 at 1 year and 14 at 10 years, demonstrating substantial clinical benefit.

### 2.8. Specific Liver Complications (Table 2)

SGLT2 inhibitor therapy reduced rates of hepatic decompensation events:

- Ascites: 0.39% vs. 0.78% at 10 years (HR 0.50, 95% CI 0.40–0.63, *p* < 0.001).
- Encephalopathy: 0.45% vs. 1.15% at 10 years (HR 0.42, 95% CI 0.34–0.52, *p* < 0.001).
- Varices: 0.25% vs. 0.35% at 10 years (HR 0.73, 95% CI 0.52–1.02, *p* = 0.064).

Notably, there was no significant difference in hepatocellular carcinoma incidence (0.36% vs. 0.46% at 10 years, *p* = 0.051) or liver transplantation rates (0.08% vs. 0.08%, *p* = 0.414).

Similarly, the need for medications to treat cirrhosis complications (ammonia-lowering agents, diuretics, and NSBB) was significantly lower in the SGLT2 inhibitor group (*p* < 0.001).

### 2.9. Metabolic Profile (Table 2)

SGLT2 inhibitor therapy resulted in significant metabolic improvements. Mean BMI decreased from a baseline of 34.0 ± 6.4 to 33.2 kg/m^2^ at 1 year in the SGLT2 inhibitor group, while remaining stable in controls (*p* < 0.001). Glycemic control improved substantially, with HbA1c of 7.35 ± 1.51% versus 7.93 ± 1.72% at 1 year (*p* < 0.001). The proportion of patients with poor metabolic markers decreased from baseline, being 36.45% in the SGLT2 inhibitor group versus 43.85% in controls at 1 year (*p* < 0.001). Differences in cumulative incidence were significant at all time points (*p* < 0.001 for each category over 10 years).

### 2.10. Liver Function Parameters (Table 2, Figure 4)

SGLT2 inhibitor therapy improved liver enzyme profiles. At 1 year, mean ALT was 40.3 ± 31.5 U/L in the SGLT2 inhibitor group versus 48.3 ± 41.2 U/L in controls (*p* < 0.001). Similar improvements were seen for AST (30.6 ± 24.8 vs. 36.4 ± 35.1 U/L, *p* < 0.001). The proportion with elevated ALT > 50 U/L was 21.70% versus 29.10% at 1 year (*p* < 0.001). ALP and total bilirubin followed the same pattern. Albumin, INR, and total bilirubin as markers of synthetic and detoxification functions were better preserved in the SGLT2 inhibitor group, with albumin <2.8 g/dL in only 0.75% of SGLT2 inhibitor patients versus 2.20% of controls at 1 year (*p* < 0.001). Thrombocytopenia as a laboratory marker for portal hypertension was significantly better preserved in the SGLT2 inhibitor group.

## 3. Discussion

This large real-world cohort study, encompassing 39,844 propensity-matched MASLD patients followed for up to 10 years, demonstrates that SGLT2 inhibitor therapy is associated with profound improvements in survival, liver-related outcomes, besides cardiovascular events. The magnitude of benefit observed—a 72% reduction in all-cause mortality and 52% reduction in advanced liver disease—represents a substantial clinical impact that exceeds many currently available therapies for MASLD.

The survival benefit observed with SGLT2 inhibitors (HR 0.28, 95% CI 0.24–0.33) is particularly striking and consistent with the cardiovascular outcome trials in diabetic populations [23,24,25]. The EMPA-REG OUTCOME trial demonstrated a 32% reduction in all-cause mortality with empagliflozin [23], while our study shows even greater benefits in the MASLD population. This enhanced benefit may reflect the combined hepatoprotective and cardiometabolic effects of SGLT2 inhibitors in a population at high risk for both liver and cardiovascular complications.

Our findings regarding liver-specific outcomes align with and extend previous clinical trials and meta-analyses. Recent systematic reviews have shown that SGLT2 inhibitors reduce liver fat content by approximately 20% and improve liver enzymes in MASLD patients [21,22]. Our real-world data demonstrate that these biochemical improvements translate into meaningful clinical outcomes, with significant reductions in hepatic decompensation events, need for liver-specific medications, and progression to advanced liver disease.

The mechanisms underlying SGLT2 inhibitors’ hepatoprotective effects are multifaceted and complementary [27]. Direct hepatic effects include enhanced fatty acid oxidation, reduced de novo lipogenesis, decreased inflammation through suppression of NF-κB and NLRP3 inflammasome pathways, and improved mitochondrial function [28,29]. Recent studies have shown that SGLT2 inhibitors modulate macrophage polarization from pro-inflammatory M1 to anti-inflammatory M2 phenotype, contributing to reduced hepatic inflammation and fibrosis [30]. Additionally, the systemic metabolic improvements—including weight loss, improved insulin sensitivity, and reduced glucotoxicity—create a favorable metabolic environment for liver health [31]. However, our study lacks direct mechanistic data such as liver histology or specific biomarkers (e.g., cytokeratin-18 fragments) that could confirm the anti-inflammatory and antifibrotic effects observed in preclinical studies.

The consistent benefits observed across different definitions of advanced liver disease (clinical, laboratory, and medication-based) strengthen the evidence for SGLT2 inhibitors’ efficacy. This consistency suggests that the benefits are not limited to surrogate markers but extend to clinically meaningful outcomes. The reduction in need for medications specific to portal hypertension (ammonia-lowering agents, diuretics, non-selective beta-blockers) indicates that SGLT2 inhibitors may delay or prevent the development of clinically significant portal hypertension.

Comparison with other MASLD therapies highlights the potential positioning of SGLT2 inhibitors in the treatment algorithm. While lifestyle modifications remain the cornerstone of MASLD management, achieving and maintaining significant weight loss is challenging [10]. Pioglitazone has shown efficacy in improving liver histology but is associated with weight gain and other adverse effects [32]. The recently approved resmetirom demonstrated histological improvements in clinical trials but lacks the extensive cardiovascular outcome data available for SGLT2 inhibitors [12]. GLP-1 receptor agonists, particularly semaglutide, have shown promising results in MASLD, and future studies should explore potential synergistic effects with SGLT2 inhibitors [33]. Direct head-to-head trials comparing SGLT2 inhibitors with GLP-1 receptor agonists and pioglitazone in MASLD populations are urgently needed to establish optimal treatment algorithms.

The cardiovascular benefits observed in our study (HR 0.86, 95% CI 0.82–0.90) are particularly relevant given the high cardiovascular risk in MASLD patients. The dual benefit of improving both liver and cardiovascular outcomes positions SGLT2 inhibitors as potentially transformative therapies for MASLD patients, particularly those with coexisting T2DM and cardiovascular risk factors. The delay in cardiovascular benefit manifestation (becoming significant only after 5 years) suggests that early initiation may be crucial for maximizing long-term benefits.

Our study’s strengths include its large sample size, extensive propensity matching, real-world setting, and unprecedented 10-year follow-up period. The use of the TriNetX platform provides access to diverse patient populations and comprehensive electronic health records, enhancing generalizability. The consistent findings across multiple outcome domains and time points provide robust evidence for SGLT2 inhibitors’ benefits in MASLD.

Several limitations warrant consideration. As with all observational studies, residual confounding cannot be completely eliminated despite extensive matching. The TriNetX database lacks detailed information on lifestyle factors, medication adherence, and specific SGLT2 inhibitor doses. We did not have access to liver biopsy data or systematic non-invasive assessment of fibrosis, relying instead on clinical outcomes and laboratory parameters. The definition of MASLD was based on ICD coding rather than systematic exclusion of other etiologies, though our exclusion criteria attempted to address this limitation.

Additionally, unmeasured factors, including lifestyle modifications, dietary patterns, physical activity levels, and baseline fibrosis stage, could not be accounted for in our analysis. Future studies should incorporate these variables through detailed questionnaires and systematic fibrosis assessment using elastography or biopsy data.

Future research should focus on several key areas. Head-to-head comparisons of different SGLT2 inhibitors may identify agent-specific benefits. Combination therapy studies, particularly with GLP-1 receptor agonists, could explore potential synergistic effects. Mechanistic studies should further elucidate the hepatoprotective pathways of SGLT2 inhibitors. Cost-effectiveness analyses are needed to support broader implementation in clinical practice. Subgroup analyses stratified by baseline fibrosis stage, diabetes status, and genetic polymorphisms (particularly PNPLA3, TM6SF2, and HSD17B13 variants) could identify patients most likely to benefit from SGLT2 inhibitor therapy. Finally, studies in MASLD patients without diabetes would help establish whether benefits extend beyond the diabetic population.

## 4. Materials and Methods

### 4.1. Data Source

This retrospective cohort study utilized real-world data from the TriNetX global health research platform, as of 30 December 2023. TriNetX includes de-identified longitudinal electronic health records from over 135 million patients across 112 healthcare organizations worldwide, encompassing hospitals, primary care clinics, and specialty centers [34]. The database includes demographic information, diagnoses (ICD-9/10), procedures, medications (orders, prescriptions, and administrations), laboratory test results (LOINC codes), and healthcare utilization. The study involved analysis of existing de-identified records, but not the raw data, and therefore was exempt from Institutional Review Board approval. This study adhered to CIOMS guidelines for ethical research involving human data, ensuring respect for autonomy and privacy through use of de-identified records only. No informed consent was required due to the retrospective, de-identified nature of the data. The study was approved by the Hadassah Medical Organization Ethics Committee. Per the TriNetX Data Use Agreement (DUA), all data are aggregated and de-identified to prevent re-identification, with no access to individual patient records and compliance with HIPAA and GDPR standards.

### 4.2. Study Population and Cohort Definitions

**Figure 5:**
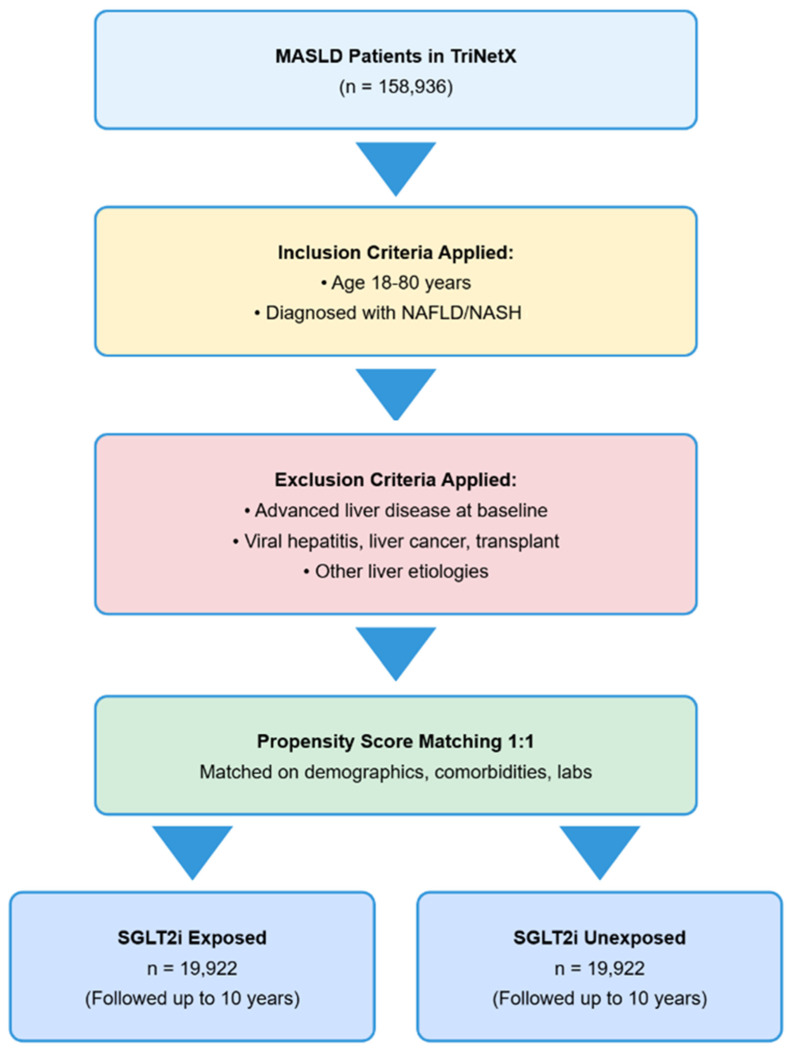
**Study Flow Diagram.** This figure illustrates the patient selection process for the propensity score-matched analysis of SGLT2 inhibitor therapy in MASLD patients. The diagram shows the initial cohort identification, application of inclusion and exclusion criteria, and final matched cohort of 19,922 patients analyzed for SGLT2 inhibitor exposure effects.

Eligible patients were adults (18–80 years) diagnosed with metabolic dysfunction-associated steatotic liver disease (MASLD) according to ICD-9/10 criteria. As the newly developed MASLD nomenclature is not available yet in the ICD-9/10 system, both non-alcoholic fatty liver disease (NAFLD) and non-alcoholic steatohepatitis are combined as MASLD. The study population included patients who had adequate follow-up data; the study flow is illustrated in Figure 5.

Two cohorts were defined based on SGLT2 inhibitor exposure. The **SGLT2 inhibitor-exposed group** comprised patients prescribed any SGLT2 inhibitor (empagliflozin, dapagliflozin, canagliflozin, or ertugliflozin) at or before the index date, with continued exposure throughout the follow-up period. All approved doses were included, administered via oral formulation. On the other hand, the **SGLT2 inhibitor-unexposed group** encompassed patients with no SGLT2 inhibitor prescriptions at baseline or during the follow-up period. This control group received standard of care for diabetes, which could include other glucose or lipid-lowering agents as well as antihypertensive medications. Both groups received standard of care for MASLD.

### 4.3. Exclusion Criteria

Patients were excluded for any of the following: (1) ALT > 4 × UNL or ALP > 4 × UNL at or before index date (baseline point), to avoid confounding from acute liver injuries or cholestatic obstructive disorders; (2) advanced liver disease, defined by diagnoses of cirrhosis or cirrhosis complications at baseline; (3) history of liver transplantation or hepatocellular carcinoma, as these were study outcomes; (4) use of anticoagulants, to preserve INR evaluation integrity; (5) coexisting chronic liver diseases of non-metabolic etiologies, including viral hepatitis, autoimmune hepatitis, primary biliary cholangitis, primary sclerosing cholangitis, Wilson’s disease, hemochromatosis, or alpha-1 antitrypsin deficiency.

Appendix A.1 summarizes the cohort definitions and TriNetX codes of the inclusion and exclusion criteria.

### 4.4. Propensity Score Matching

Propensity scores were calculated using logistic regression incorporating 34 covariates, including demographics (age, sex, race), comorbidities (diabetes, hypertension, cardiovascular conditions), laboratory values (liver enzymes, metabolic parameters), and medications. These covariates were drawn from the 12 months preceding the index date to reflect a contemporaneous clinical profile.

A 1:1 greedy matching algorithm (without replacement) was applied using a standard caliper width, resulting in 19,922 matched pairs. Covariate balance was assessed using standardized mean differences (St. Diff.), with <0.3 considered acceptable. *p*-values were interpreted only when St. Diff. ≥ 0.3.

### 4.5. Outcomes

Outcomes were assessed at 1, 5, and 10 years following the index date and included:

- Primary outcomes: all-cause mortality and overall survival.
- Liver-related outcomes: advanced liver disease (ALD) was defined according to clinical diagnoses (e.g., portal hypertension, ascites, varices, hepatic encephalopathy), laboratory abnormalities (e.g., thrombocytopenia, hyperbilirubinemia, hypoalbuminemia, hyperammonemia), or dispensed medications used specifically for cirrhosis (e.g., propranolol, lactulose, rifaximin, spironolactone).
- Cardiovascular events: myocardial infarction, stroke, atrial fibrillation, and heart failure.
- Metabolic parameters: LDL, HDL, triglycerides, HbA1c, BMI.
- Disease progression: changes in liver enzymes, liver synthetic function, and development of liver-related complications.

Composite outcomes were created using a combination of clinical diagnoses, lab abnormalities, and relevant medication use. Detailed coding algorithms for outcome definitions are provided in Appendix A.1 and Appendix A.2.

### 4.6. Statistical Analysis

Kaplan–Meier curves were used to evaluate survival and mortality over time. Hazard ratios (HRs) with 95% confidence intervals (CIs) were calculated using Cox proportional hazards models adjusted for relevant baseline variables. The number needed to treat (NNT) to prevent one clinical outcome was calculated as the reciprocal of the absolute risk reduction.

Continuous variables were compared using *t*-tests, and categorical variables using chi-square tests. Results are expressed as means ± standard deviations or proportions. Subgroup analyses were conducted to identify patient characteristics associated with differential treatment effects. A two-sided *p*-value < 0.05 was considered statistically significant.

## 5. Conclusions

Our study provides compelling real-world evidence that SGLT2 inhibitor therapy in MASLD patients is associated with substantial reductions in mortality, advanced liver disease, and cardiovascular events over 10 years of follow-up. These findings support the potential role of SGLT2 inhibitors as disease-modifying therapy in MASLD, particularly for patients with metabolic comorbidities. Given the limited therapeutic options currently available for MASLD and the favorable safety profile of SGLT2 inhibitors, these agents warrant consideration in the management of MASLD patients, especially those with T2DM or high cardiovascular risk.

## Figures and Tables

**Figure 3:**
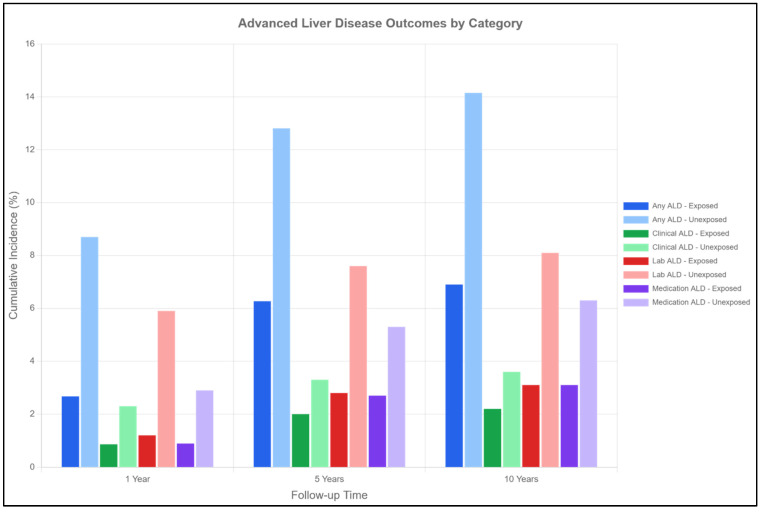
Advanced Liver Disease Outcomes by Category. This grouped bar chart illustrates the cumulative incidence (%) of advanced liver disease (ALD) outcomes at 1, 5, and 10 years of follow-up. Six categories are shown: Any ALD (composite), Clinical ALD (diagnoses of portal hypertension, varices, ascites, encephalopathy), Laboratory ALD (thrombocytopenia, hyperbilirubinemia, hypoalbuminemia), and Medication ALD (use of lactulose, rifaximin, beta-blockers, or spironolactone for liver complications). Blue bars represent SGLT2-exposed patients; red bars represent unexposed patients. At 10 years, the incidence of any ALD was 6.9% in SGLT2-exposed versus 14.2% in unexposed patients. All between-group differences were statistically significant (*p* < 0.001) at each time point for every ALD category, demonstrating consistent protective effects of SGLT2 inhibitor therapy across all definitions of advanced liver disease.

**Figure 4:**
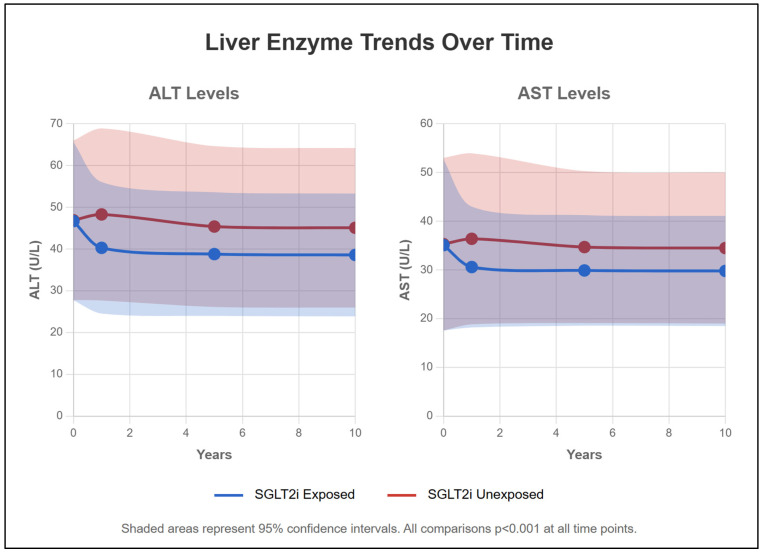
Liver Enzyme Trends Over Time. This figure displays temporal trends in liver enzymes (ALT and AST) over the 10-year follow-up period using line graphs with 95% confidence interval bands (shaded areas). The blue lines represent SGLT2 inhibitor-exposed patients, while red lines represent unexposed patients. Points on the lines indicate mean values at baseline, 1, 5, and 10 years. Both groups showed improvement from baseline, with SGLT2-exposed patients maintaining consistently lower enzyme levels throughout follow-up. The shaded areas represent 95% confidence intervals, with non-overlapping bands indicating statistically significant differences between groups (*p* < 0.001 at all time points). Normal ranges: ALT < 40 U/L, AST < 40 U/L.

**Table 1:** Baseline Demographics, Comorbidities, and Laboratory Parameters (After Propensity Matching). (**a**) Demographics and Comorbidities. (**b**) Baseline Laboratory Parameters.

(**a**)				
**Characteristic**	**SGLT2i Group**	**Non-SGLT2i Group**	***p*** **-Value**	**St. Diff.**
	**(n = 19,922)**	**(n = 19,922)**		
**Demographics**				
**Age (years), mean ± SD**	55.5 ± 11.8	55.6 ± 11.9	0.87	0.0084
**Female, n (%)**	9647 (48.4%)	9644 (48.4%)	0.94	0.0003
**Race (%)**				
**- White**	12,044 (60.5%)	12,051 (60.5%)	0.91	0.0014
**- Black**	1709 (8.6%)	1706 (8.6%)	0.93	0.0009
**- Asian**	1926 (9.7%)	1931 (9.7%)	0.91	0.0013
**BMI (kg/m^2^), mean ± SD**	34.0 ± 6.4	34.1 ± 6.5	0.76	0.0156
**Comorbidities (%)**				
**- Diabetes mellitus**	18,008 (90.4%)	18,015 (90.4%)	0.91	0.0011
**- Hypertension**	13,291 (66.7%)	13,297 (66.7%)	0.93	0.0008
**- Ischemic heart disease**	2202 (11.1%)	2198 (11.0%)	0.91	0.0012
**- Cerebrovascular disease**	525 (2.6%)	522 (2.6%)	0.89	0.0019
(**b**)				
**Laboratory Parameter**	**SGLT2i Group**	**Non-SGLT2i Group**	***p*** **-Value**	**St. Diff.**
	
**Liver function tests**				
**ALT (U/L), mean ± SD**	46.7 ± 37.9	46.9 ± 38.2	0.89	0.0052
	(n = 14,743, 74%)	(n = 14,751, 74%)		
**AST (U/L), mean ± SD**	35.1 ± 35.1	35.3 ± 35.4	0.91	0.0057
	(n = 14,423, 72%)	(n = 14,418, 72%)		
**ALP (U/L), mean ± SD**	84.8 ± 35.9	85.1 ± 36.2	0.88	0.0083
	(n = 13,806, 69%)	(n = 13,812, 69%)		
**Gamma-Glutamyl Transferase (GGT) (U/L), mean ± SD**	95.5 ± 178.4	96.2 ± 180.1	0.93	0.0039
	(n = 981, 4.9%)	(n = 978, 4.9%)		
**Total bilirubin (mg/dL), mean ± SD**	0.60 ± 0.30	0.61 ± 0.31	0.87	0.0328
	(n = 13,745, 69%)	(n = 13,751, 69%)		
**Albumin (g/dL), mean ± SD**	4.30 ± 0.40	4.29 ± 0.41	0.91	0.0248
	(n = 13,847, 70%)	(n = 13,843, 69%)		
**Metabolic parameters**				
**HbA1c (%), mean ± SD**	8.00 ± 1.70	8.02 ± 1.72	0.88	0.0117
	(n = 13,934, 70%)	(n = 13,928, 70%)		
**Total cholesterol (mg/dL), mean ± SD**	170.0 ± 47.8	170.4 ± 48.1	0.92	0.0083
	(n = 12,500, 63%)	(n = 12,495, 63%)		
**LDL (mg/dL), mean ± SD**	89.8 ± 36.9	90.1 ± 37.2	0.9	0.0081
	(n = 12,312, 62%)	(n = 12,308, 62%)		
**HDL (mg/dL), mean ± SD**	41.5 ± 14.5	41.4 ± 14.6	0.94	0.0069
	(n = 12,670, 64%)	(n = 12,665, 64%)		
**Triglycerides (mg/dL), mean ± SD**	210.3 ± 207.0	211.5 ± 208.8	0.88	0.0058
	(n = 12,567, 63%)	(n = 12,562, 63%)		
**Hematologic parameters**				
**Platelet count (×10^3^/μL), mean ± SD**	256.9 ± 71.0	257.4 ± 71.5	0.91	0.007
	(n = 12,087, 61%)	(n = 12,092, 61%)		
**INR, mean ± SD**	1.00 ± 0.10	1.01 ± 0.11	0.89	0.0953
	(n = 1867, 9.4%)	(n = 1863, 9.3%)		
**Other markers**				
**AFP (ng/mL), mean ± SD**	3.20 ± 1.50	3.22 ± 1.52	0.93	0.0133
	(n = 95, 0.5%)	(n = 93, 0.5%)		
**Creatinine (mg/dL), mean ± SD**	0.90 ± 1.90	0.91 ± 1.92	0.9	0.0052
	(n = 15,129, 76%)	(n = 15,124, 76%)		

(**a**) Legend: This table presents the baseline demographic and comorbidity data for the propensity score-matched SGLT2 inhibitor-exposed and SGLT2 inhibitor-unexposed MASLD cohorts. *p*-values > 0.05 and standardized differences (St. Diff.). SGLT2i = SGLT2 inhibitor. (**b**) Legend: This table presents the baseline laboratory parameters for the propensity score-matched SGLT2 inhibitor-exposed and SGLT2 inhibitor-unexposed MASLD cohorts. Lab availability varied; percentages indicate proportion of patients with at least one measurement in the year before index. Values represent means ± standard deviations for patients with measurements available. *p*-values from *t*-test for means, with standardized mean difference (St. Diff.). SGLT2i = SGLT2 inhibitor.

**Table 2:** (**a**) Impact of SGLT2 Inhibitors on Clinical Outcomes over 1-, 5-, and 10-Years Follow-up. (**b**) Laboratory Outcomes—Continuous Variables. (**c**) Laboratory Outcomes—Categorical Variables.

(**a**)						
**Clinical Outcomes**	**SGLT2i Status**	**1 Year**	**5 Years**	**10 Years**	**HR (95% CI)**	***p*** **-Value**
**Mortality**						
	Exposed	0.30%	0.87%	1.06%	0.28 (0.24–0.33)	<0.001
	Unexposed	1.63%	3.21%	3.75%		
	*p*-value	<0.001	<0.001	<0.001		
**Survival**						
	Exposed	99.64%	98.11%	95.00%	-	-
	Unexposed	98.11%	94.24%	88.69%		
	*p*-value	<0.001	<0.001	<0.001		
**Cardiovascular events**						
	Exposed	5.99%	9.50%	10.19%	0.86 (0.82–0.90)	<0.001
	Unexposed	6.19%	10.28%	11.80%		
	*p*-value	0.390	0.009	<0.001		
**Advanced liver disease**						
	Exposed	2.67%	6.27%	6.90%	0.48 (0.45–0.51)	<0.001
	Unexposed	8.70%	12.81%	14.15%		
	*p*-value	<0.001	<0.001	<0.001		
**ALD (clinical)**						
	Exposed	0.87%	2.00%	2.20%	0.61 (0.54–0.68)	<0.001
	Unexposed	2.30%	3.30%	3.60%		
	*p*-value	<0.001	<0.001	<0.001		
**ALD (laboratory)**						
	Exposed	1.20%	2.80%	3.10%	0.38 (0.35–0.42)	<0.001
	Unexposed	5.90%	7.60%	8.10%		
	*p*-value	<0.001	<0.001	<0.001		
**ALD (medications)**						
	Exposed	0.90%	2.70%	3.10%	0.49 (0.44–0.54)	<0.001
	Unexposed	2.90%	5.30%	6.30%		
	*p*-value	<0.001	<0.001	<0.001		
**Liver transplantation**						
	Exposed	0.01%	0.06%	0.08%	1.00 (0.50–2.00)	1.000
	Unexposed	0.05%	0.06%	0.08%		
	*p*-value	0.002	0.670	0.414		
**Hepatocellular carcinoma**						
	Exposed	0.13%	0.32%	0.36%	0.78 (0.60–1.02)	0.070
	Unexposed	0.17%	0.41%	0.46%		
	*p*-value	0.018	0.035	0.051		
**Ascites**						
	Exposed	0.15%	0.35%	0.39%	0.50 (0.40–0.63)	<0.001
	Unexposed	0.43%	0.69%	0.78%		
	*p*-value	<0.001	<0.001	<0.001		
**Encephalopathy**						
	Exposed	0.15%	0.35%	0.45%	0.42 (0.34–0.52)	<0.001
	Unexposed	0.60%	0.95%	1.15%		
	*p*-value	<0.001	<0.001	<0.001		
**Varices**						
	Exposed	0.08%	0.18%	0.25%	0.73 (0.52–1.02)	0.064
	Unexposed	0.16%	0.25%	0.35%		
	*p*-value	<0.001	0.017	0.189		
**Ammonia lowering agents**						
	Exposed	1.15%	2.95%	3.40%	0.52 (0.47–0.57)	<0.001
	Unexposed	2.90%	5.75%	6.65%		
	*p*-value	<0.001	<0.001	<0.001		
**Diuretics**						
	Exposed	3.65%	8.00%	8.85%	0.68 (0.64–0.72)	<0.001
	Unexposed	6.20%	11.90%	13.20%		
	*p*-value	<0.001	<0.001	<0.001		
**NSBB**						
	Exposed	0.70%	2.05%	2.35%	0.55 (0.49–0.62)	<0.001
	Unexposed	1.65%	3.75%	4.35%		
	*p*-value	<0.001	<0.001	<0.001		
(**b**)						
**Laboratory Parameter**	**SGLT2i Status**	**1 Year**	**5 Years**	**10 Years**	***p*** **-Value (10 Years)**	
**ALT (U/L)**						
	Exposed	40.3 ± 31.5	38.8 ± 29.6	38.6 ± 29.4	<0.001	
	Unexposed	48.3 ± 41.2	45.4 ± 38.5	45.1 ± 38.2		
	*p*-value	<0.001	<0.001	<0.001		
**AST (U/L)**						
	Exposed	30.6 ± 24.8	29.9 ± 22.7	29.8 ± 22.6	<0.001	
	Unexposed	36.4 ± 35.1	34.7 ± 31.2	34.5 ± 31.0		
	*p*-value	<0.001	<0.001	<0.001		
**ALP (U/L)**						
	Exposed	81.4 ± 33.2	80.8 ± 32.5	80.7 ± 32.4	<0.001	
	Unexposed	86.9 ± 38.7	85.6 ± 37.1	85.4 ± 36.9		
	*p*-value	<0.001	<0.001	<0.001		
**Total bilirubin (mg/dL)**						
	Exposed	0.56 ± 0.29	0.57 ± 0.30	0.57 ± 0.30	<0.001	
	Unexposed	0.63 ± 0.40	0.65 ± 0.43	0.66 ± 0.44		
	*p*-value	<0.001	<0.001	<0.001		
**Albumin (g/dL)**						
	Exposed	4.27 ± 0.41	4.26 ± 0.41	4.26 ± 0.41	<0.001	
	Unexposed	4.20 ± 0.46	4.18 ± 0.47	4.17 ± 0.47		
	*p*-value	<0.001	<0.001	<0.001		
**Platelet count (×10^3^/μL)**						
	Exposed	255.8 ± 68.2	253.4 ± 66.5	251.9 ± 65.8	<0.001	
	Unexposed	249.3 ± 72.8	244.8 ± 70.5	241.2 ± 69.3		
	*p*-value	<0.001	<0.001	<0.001		
**HbA1c (%)**						
	Exposed	7.35 ± 1.51	7.28 ± 1.47	7.26 ± 1.46	<0.001	
	Unexposed	7.93 ± 1.72	7.88 ± 1.69	7.86 ± 1.68		
	*p*-value	<0.001	<0.001	<0.001		
**BMI (kg/m^2^)**						
	Exposed	33.2 ± 6.2	32.8 ± 6.1	32.7 ± 6.1	<0.001	
	Unexposed	34.1 ± 6.5	33.9 ± 6.4	33.8 ± 6.4		
	*p*-value	<0.001	<0.001	<0.001		
**Creatinine (mg/dL)**						
	Exposed	0.91 ± 0.82	0.93 ± 0.85	0.94 ± 0.86	0.026	
	Unexposed	0.96 ± 1.24	0.99 ± 1.31	1.01 ± 1.35		
	*p*-value	<0.001	<0.001	0.026		
(**c**)						
**Categorical Outcome**	**SGLT2i Status**	**1 Year**	**5 Years**	**10 Years**	***p*** **-Value (10 Years)**	
**ALT > 50 U/L**						
	Exposed	21.70%	25.00%	25.15%	<0.001	
	Unexposed	29.10%	34.80%	35.60%		
	*p*-value	<0.001	<0.001	<0.001		
**AST > 40 U/L**						
	Exposed	13.85%	15.70%	15.85%	<0.001	
	Unexposed	19.40%	22.35%	22.90%		
	*p*-value	<0.001	<0.001	<0.001		
**Bilirubin ≥ 2 mg/dL**						
	Exposed	0.65%	0.90%	0.95%	<0.001	
	Unexposed	1.70%	2.35%	2.55%		
	*p*-value	<0.001	<0.001	<0.001		
**Albumin ≤ 2.8 g/dL**						
	Exposed	0.75%	1.05%	1.10%	<0.001	
	Unexposed	2.20%	2.95%	3.20%		
	*p*-value	<0.001	<0.001	<0.001		
**INR ≥ 1.7**						
	Exposed	0.30%	0.50%	0.60%	<0.001	
	Unexposed	0.75%	1.20%	1.45%		
	*p*-value	<0.001	<0.001	<0.001		
**Platelets < 150 × 10^3^/μL**						
	Exposed	7.15%	8.65%	9.05%	<0.001	
	Unexposed	11.45%	14.05%	14.95%		
	*p*-value	<0.001	<0.001	<0.001		
**Platelets < 100 × 10^3^/μL**						
	Exposed	1.80%	2.20%	2.35%	<0.001	
	Unexposed	3.75%	4.65%	5.00%		
	*p*-value	<0.001	<0.001	<0.001		
**HbA1c > 8.5%**						
	Exposed	24.50%	27.30%	27.60%	<0.001	
	Unexposed	33.20%	38.45%	39.35%		
	*p*-value	<0.001	<0.001	<0.001		
**Poor metabolic markers**						
	Exposed	36.45%	41.20%	41.65%	<0.001	
	Unexposed	43.85%	50.70%	51.80%		
	*p*-value	<0.001	<0.001	<0.001		

(**a**) Legend: This table summarizes the effects of SGLT2 inhibitor therapy on key clinical outcomes in MASLD patients over 1, 5, and 10 years of follow-up. The outcomes include mortality, survival, cardiovascular events, advanced liver disease (ALD) manifestations, liver transplantation, hepatocellular carcinoma, and liver-related complications. HR = hazard ratio; CI = confidence interval; NSBB = non-selective beta-blockers. SGLT2i = SGLT2 inhibitor. (**b**) Legend: This table presents the mean values and standard deviations of various laboratory parameters in MASLD patients with and without SGLT2 inhibitor exposure over 1, 5, and 10 years of follow-up. SGLT2i = SGLT2 inhibitor. (**c**) Legend: This table shows the percentage of MASLD patients meeting specific categorical laboratory criteria in the SGLT2 inhibitor-exposed and unexposed groups over 1, 5, and 10 years of follow-up. Poor metabolic markers defined as LDL ≥ 130 mg/dL, HDL ≤ 40 mg/dL, triglycerides ≥ 200 mg/dL, or HbA1c ≥ 8%. SGLT2i = SGLT2 inhibitor.

**Table 3:** Number Needed to Treat to Prevent One Clinical Outcome at Different Time Points.

Outcome	NNT at 1 Year	NNT at 5 Years	NNT at 10 Years
**All-cause mortality**	74	43	36
**Advanced liver disease (any)**	17	16	14
**Clinical ALD manifestations**	70	77	71
**Laboratory-defined ALD**	21	21	20
**ALD requiring medications**	50	39	31
**Cardiovascular event**	500	125	63

Legend: This table shows the number needed to treat (NNT) with SGLT2 inhibitors to prevent one clinical event at different time points. NNT was calculated as the reciprocal of the absolute risk reduction. Lower NNT indicates greater treatment efficacy. ALD = advanced liver disease.

## Data Availability

The data that support the findings of this study are not publicly available due to restrictions from the TriNetX Data Use Agreement, which prohibits sharing of raw patient data to protect patient privacy and confidentiality. Data access is limited to aggregated, de-identified results as presented in this manuscript.

## Appendix A

### Appendix A.1. TriNetX Definitions and Codes

**Inclusion Criteria**

- **Age**: Between 18 and 80 years (TNX:9074).
- **MASLD diagnosis**:

○ Nonalcoholic steatohepatitis (UMLS:ICD10CM:K75.81).
○ Fatty liver (UMLS:ICD10CM:K76.0).

- **SGLT2 inhibitor exposure:**

○ Empagliflozin (NLM:RXNORM:1545653).
○ Dapagliflozin (NLM:RXNORM:1488564).
○ Canagliflozin (NLM:RXNORM:1373458).
○ Ertugliflozin (NLM:RXNORM:1992672).

**Exclusion Criteria**

- **Advanced liver disease at baseline**: No anticoagulants, esophageal varices, ascites, encephalopathy, medications for liver complications, hepatomegaly, liver fibrosis/cirrhosis, or abnormal laboratory values prior to index date.
- **Liver cancer/transplantation**: No liver transplantation procedures, liver cell carcinoma, or liver transplant status.
- **Viral hepatitis**: No hepatitis B or C diagnosis.
- **Other liver etiologies**: No other causes of liver disease (primary sclerosing cholangitis, primary biliary cirrhosis, hemochromatosis, etc.).

### Appendix A.2. Outcome Definitions

**Clinical Outcomes**

- **Mortality**: Deceased status.
- **Cardiovascular events**: Cerebrovascular disease (ICD10:I60-I69), myocardial infarction (ICD10:I21-I24), heart failure (ICD10:I50), or atrial fibrillation/flutter (ICD10:I48).
- **Advanced liver disease (clinical)**: Portal hypertension (ICD10:K76.6), ascites (ICD10:R18), encephalopathy (ICD10:G93.40), hepatorenal syndrome (ICD10:K76.7), hepatopulmonary syndrome (ICD10:K76.81), spontaneous bacterial peritonitis (ICD10:K65.2), varices (ICD10:I85, I86.4), liver failure (ICD10:K72.9), or cirrhosis (ICD10:K74.6).
- **Advanced liver disease (laboratory)**: Platelets ≤ 100 × 10^3^/μL (TNX:9020), bilirubin ≥ 2 mg/dL (TNX:9050), albumin ≤ 2.8 g/dL (TNX:9045), or ammonia ≥ 50 μmol/L (TNX:LG4629-4).
- **Advanced liver disease (medications)**: Neomycin (RXNORM:7299), rifaximin (RXNORM:35619), propranolol (RXNORM:8787), carvedilol (RXNORM:20352), or high-dose spironolactone (RXNORM:9997).

**Laboratory Outcomes**

- **Liver inflammatory markers**: ALT ≥ 50 U/L (TNX:9044), AST ≥ 50 U/L (TNX:9047), ALP ≥ 80 U/L (TNX:9046), or GGT ≥ 80 U/L (TNX:9051).
- **Synthetic impairment**: Bilirubin ≥ 2 mg/dL (TNX:9050), albumin ≤ 2.8 g/dL (TNX:9045), or INR ≥ 1.7 (TNX:9032).
- **Poor metabolic markers**: LDL ≥ 130 mg/dL (TNX:9002), HDL ≤ 40 mg/dL (TNX:9001), triglycerides ≥ 200 mg/dL (TNX:9004), or HbA1c ≥ 8% (TNX:9037).
